# The Spectrum of *SPTA1*-Associated Hereditary Spherocytosis

**DOI:** 10.3389/fphys.2019.00815

**Published:** 2019-07-03

**Authors:** Satheesh Chonat, Mary Risinger, Haripriya Sakthivel, Omar Niss, Jennifer A. Rothman, Loan Hsieh, Stella T. Chou, Janet L. Kwiatkowski, Eugene Khandros, Matthew F. Gorman, Donald T. Wells, Tamara Maghathe, Neha Dagaonkar, Katie G. Seu, Kejian Zhang, Wenying Zhang, Theodosia A. Kalfa

**Affiliations:** ^1^Department of Pediatrics, Emory University School of Medicine, Atlanta, GA, United States; ^2^Aflac Cancer and Blood Disorders Center, Children’s Healthcare of Atlanta, Atlanta, GA, United States; ^3^College of Nursing, University of Cincinnati, Cincinnati, OH, United States; ^4^Cancer and Blood Diseases Institute, Cincinnati Children’s Hospital Medical Center, Cincinnati, OH, United States; ^5^Department of Pediatrics, University of Cincinnati College of Medicine, Cincinnati, OH, United States; ^6^Duke University Medical Center, Durham, NC, United States; ^7^Division of Hematology, CHOC Children’s Hospital and UC Irvine Medical Center, Orange, CA, United States; ^8^Division of Hematology, Children’s Hospital of Philadelphia, Philadelphia, PA, United States; ^9^Department of Pediatrics, Perelman School of Medicine, University of Pennsylvania, Philadelphia, PA, United States; ^10^Kaiser Permanente Santa Clara Medical Center, Santa Clara, CA, United States; ^11^Dell Children’s Medical Center, Austin, TX, United States; ^12^Genomics Analysis Facility, Institute for Genomic Medicine, Columbia University, New York, NY, United States; ^13^Coyote Bioscience Co., Ltd., San Jose, CA, United States; ^14^Laboratory of Genetics and Genomics, Division of Human Genetics, Cincinnati Children’s Hospital Medical Center, Cincinnati, OH, United States

**Keywords:** *SPTA1*, α-spectrin, α^LEPRA^, hereditary spherocytosis, next generation sequencing, hemolytic anemia, *hydrops fetalis*

## Abstract

Hereditary spherocytosis (HS) is the most common red blood cell (RBC) membrane disorder causing hereditary hemolytic anemia. Patients with HS have defects in the genes coding for ankyrin (*ANK1*), band 3 (*SLC4A1*), protein 4.2 (*EPB42*), and α (*SPTA1*) or β-spectrin (*SPTB*). Severe recessive HS is most commonly due to biallelic *SPTA1* mutations. α-spectrin is produced in excess in normal erythroid cells, therefore *SPTA1*-associated HS ensues with mutations causing significant decrease of normal protein expression from both alleles. In this study, we systematically compared genetic, rheological, and protein expression data to the varying clinical presentation in eleven patients with *SPTA1*-associated HS. The phenotype of HS in this group of patients ranged from moderately severe to severe transfusion-dependent anemia and up to *hydrops fetalis* which is typically fatal if transfusions are not initiated before term delivery. The pathogenicity of the mutations could be corroborated by reduced *SPTA1* mRNA expression in the patients’ reticulocytes. The disease severity correlated to the level of α-spectrin protein in their RBC cytoskeleton but was also affected by other factors. Patients carrying the low expression α^LEPRA^ allele *in trans* to a null *SPTA1* mutation were not all transfusion dependent and their anemia improved or resolved with partial or total splenectomy, respectively. In contrast, patients with near-complete or complete α-spectrin deficiency have a history of having been salvaged from fatal *hydrops fetalis*, either because they were born prematurely and started transfusions early or because they had intrauterine transfusions. They have suboptimal reticulocytosis or reticulocytopenia and remain transfusion dependent even after splenectomy; these patients require either lifetime transfusions and iron chelation or stem cell transplant. Comprehensive genetic and phenotypic evaluation is critical to provide accurate diagnosis in patients with *SPTA1*-associated HS and guide toward appropriate management.

## Introduction

Hereditary spherocytosis (HS) is the most common red blood cell (RBC) cytoskeleton disorder causing hereditary hemolytic anemia (HHA), characterized by sphere-shaped erythrocytes (spherocytes) with increased osmotic fragility. HS can affect all ethnic groups but is more common in people of northern European ancestry where the prevalence is 1 in 1000–2500 (Gallagher, 2005). Spherocytes are formed because of loss of membrane due to quantitative defects in proteins that link the cytoskeleton to the lipid bilayer (“vertical” linkages) (Eber and Lux, 2004). The scaffolding network of the RBC cytoskeleton is assembled by α- and β-spectrin heterodimers self-associating in a head-to-head fashion to form tetramers, bound to the lipid membrane via the anchoring complex of ankyrin, protein 4.2, and band 3 (Salomao et al., 2008). In autosomal dominant HS, which accounts for approximately 75% of cases, mutations of ankyrin (*ANK1*), band 3 (*SLC4A1*), and β-spectrin (*SPTB*) genes predominate. Recessive HS is most often due to compound heterozygosity for defects in the genes encoding ankyrin, α-spectrin (*SPTA1*), or protein 4.2 (*EPB42*) (Eber and Lux, 2004; Gallagher, 2005; Mohandas, 2018).

Two normal *SPTA1* alleles allow for overproduction of α-spectrin chains (Hanspal and Palek, 1987). Therefore, HS due to α-spectrin deficiency manifests when both of the *SPTA1* alleles are affected by mutations causing significant quantitative defect. Two *SPTA1* low expression alleles were identified early-on to be associated with RBC membrane disorders and their study helped to determine the quantitative requirements of the RBC cytoskeleton for α-spectrin (Wilmotte et al., 1993; Wichterle et al., 1996). α^LELY^ (Low Expression LYon) has a minor allele frequency (MAF) of 25.5% (gnomad.broadinstitute.org) and consists of the mutation c.6531-12C>T in intron 45, causing partial skipping of exon 46 in half of the transcripts and consequently a 50% decrease in the amount of α-spectrin (Wilmotte et al., 1993; Marechal et al., 1995). α^LELY^ *in trans* to an *SPTA1* allele with a hereditary elliptocytosis (HE)-associated mutation modifies the phenotype from HE to hereditary pyropoikilocytosis (Niss et al., 2016). In contrast, α^LELY^ *in trans* to a null *SPTA1* allele causes no disease, indicating that production of ∼25% of normal α-spectrin is enough for normal RBC cytoskeleton assembly (Delaunay et al., 2004). α^LEPRA^ (Low Expression PRAgue) is a deep intronic *SPTA1* mutation (c.4339-99C > T). Positioned at -99 of intron 30, it activates an alternative acceptor splice site at position -70 of the same intron. The alternative splicing results in frameshift and premature termination of translation, leading to decreased α-spectrin production. This allele (MAF of 0.5% per gnomad.broadinstitute.org) produces only about 16% of full-length spectrin as compared to the normal *SPTA1* allele, based on studies with metabolic labeling of erythroblasts *in vitro* (Wichterle et al., 1996). α^LEPRA^ *in trans* to a null *SPTA1* allele (leading to a total α-spectrin production of about 8%) has been shown to cause severe autosomal recessive HS, with anemia and jaundice that resolve with splenectomy (Wichterle et al., 1996; Delaunay et al., 2004). Complete α-spectrin deficiency has been shown to cause lethal anemia *in utero* (Whitfield et al., 1991).

We present here eleven patients with HS due to α-spectrin deficiency and discuss their phenotype/genotype correlation (Table 1).

**TABLE 1:** Genetic mutations and associated phenotype in HS due to *SPTA1* mutations.

**Phenotype**	**Patient**	**Allele 1**	**Allele 2**	**Age at time of report and comments**	**Ektacytometry**	**α-spectrin in RBC ghosts (% of control)**
GROUP I (patients 1–4) Severe, recessive HS (transfusion-dependent, responding to splenectomy)	1	c.4339-99C > T	c.4295del (p.L1432^*^)	11 year-old, chronic transfusion requirement with partial response to partial splenectomy, resolved after total splenectomy	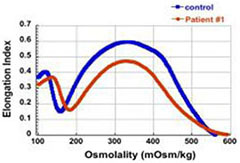	54%
	2	c.4339-99C > T	c.5102A > T (p.L1701^*^)	7 year-old, chronic transfusion requirement, improved with partial splenectomy	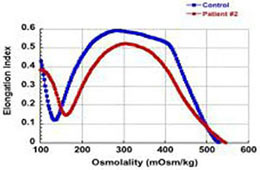	64%
	3	c.4339-99C > T	c.3267A > T (p.Y1089^*^)	11 year-old, not splenectomized due to family preference, continues to require frequent transfusions	Not evaluable in a transfused sample	
	4	Mutation not identified	Gross deletion of *SPTA1*	3.5 year-old, RT-PCR demonstrated significantly decreased α-spectrin expression; hemoglobin has normalized after recent splenectomy	Not evaluable in a transfused sample	
GROUP II (patients 5–8) Severe to moderately severe, recessive HS	5	c.4339-99C > T	c.1120C > T (p.R374^*^)	4 year-old, chronic transfusion requirement for first three years with improved pattern since.	Sample not provided after age 3, when transfusion-independent	
	6	c.4339-99C > T	c.1351-1G > T	7 year-old, occasional transfusion requirement, resolved after splenectomy at 5 years of age	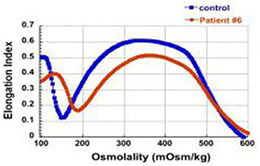	59%
	7	c.4339-99C>T	c.2671C > T (p.R891^*^)	4 year-old, has not been transfused so far, Hgb 7.1-8.9 g/dL, ARC 420-572 x 10^3^/μl.	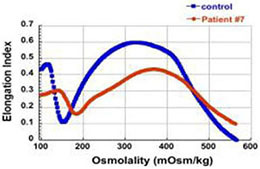	61%
	8	c.4339-99C > T	c.3257delT	8 year-old, transfused once as neonate, Hgb 10.6–11.8 g/dL, ARC 354–535 × 10^3^/μl; now Hgb 15–16 g/dL with normal ARC after splenectomy at 6 years of age (splenectomy performed because of chronic abdominal pain due to co-morbidities)	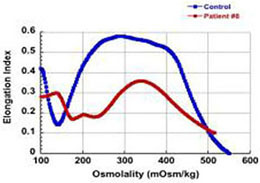	Not performed.
GROUP III (patients 9-11) Life-threatening anemia in utero leading tofatal *hydrops fetalis* if untreated (transfusion-dependent, not responding to splenectomy)	9	c.4206delG (fs)	c.4180delT (fs) in haplotype with c.6631C > T (p.R2211C)	Died at birth. Post-mortem diagnosis from parental studies and DNA extracted from liver tissue saved in paraffin block	N/A	
	10	c.6788+11C > T	c.6788+11C > T	11 year-old, born prematurely at EGA of 33 weeks with *hydrops fetalis*, remained transfusion-dependent even after splenectomy; now doing well after matched sibling transplant	Not evaluable in a transfused sample (required chronic transfusions up until bone marrow transplant)	26% (performed in CD71+ cells)
	11	c.6154del (p.Ala2052fs)	c.6154del (p.Ala2052fs)	2 year-old, severe in-utero anemia requiring five *in-utero* transfusions. Born with severe neonatal hyperbilirubinemia requiring exchange transfusion. Remains transfusion-dependent	Not evaluable in a transfused sample	

Of note, all the *SPTA1* variants reported here except c.4339-99C > T (α^*LEPRA*^) and c.2671C > T; p.R891^*^ (Bogardus et al., 2014) have not been previously described.

## Materials and Methods

### Next Generation Sequencing (NGS) of Genes Associated With HHA

Patients with the clinical diagnosis of HHA and their parents were enrolled in an Institutional Review Board-approved research protocol based at Cincinnati Children’s Hospital Medical Center. DNA was isolated from peripheral blood (in the case of patient 9 from liver tissue preserved in paraffin after autopsy), and analyzed on an NGS HHA panel; the regions of interest for enrichment and DNA sequencing included the coding exons plus 20 bases of intronic boundaries for 32 genes known to be associated with RBC membrane and enzyme disorders and with congenital dyserythropoietic anemias: *ABCG5, ABCG8, AK1, ALDOA, ANK1, C15orf41, CDAN1, EPB41, EPB42, G6PD, GATA1, GCLC, GPI, GPX1, GSR, GSS, HK1, KIF23, KLF1, NT5C3A, PFKM, PGK1, PIEZO1, PKLR, RHAG, SEC23B, SLC2A1(GLUT1), SLC4A1, SPTA1, SPTB, TPI1*, and *XK*. Regulatory regions and deep intronic areas of these genes with published disease-causing mutations were included in the HHA panel design. Sanger sequencing was used to confirm all mutations found in patients and in parental samples (except for parents of patients #7 and #11) to establish the phase.

### Osmotic Gradient Ektacytometry

Whole blood samples were collected in K2-EDTA-containing vials from study subjects at least 3 months after last transfusion to avoid misinterpretation from the presence of donor RBCs. Samples from healthy volunteers were collected at same time and shipped as travel controls. Specimens were stored or shipped at 4°C and were analyzed within 24 h of sample collection using LoRRca® MaxSis (Mechatronics, United States LLC, Warwick, RI, United States). RBC deformation was recorded while the cells were exposed to a constant shear stress of 30 Pa and an increasing osmotic gradient (0–600 mOsm/kg) in order to generate the ektacytometry curve (Da Costa et al., 2016; Zaninoni et al., 2018).

### Capillary Electrophoresis and Immunodetection

Red blood cell membrane “ghosts” were prepared from patients #1, #6, and #7, at least 3 months after last transfusion, by hypotonic lysis (Bennett, 1983) and cytoskeletal proteins were evaluated by immunodetection using the size-based capillary electrophoresis instrument Wes (ProteinSimple, San Jose, CA, United States). Capillary immuno-electrophoresis was also performed on lysates prepared from isolated CD71+ cells from patient #10 (with a similarly prepared healthy volunteer sample as control).

### Quantitative Real-Time PCR (qPCR) of Reticulocyte mRNA

Reticulocytes were magnetically isolated from whole blood collected in K2-EDTA-containing vials from patients #4 and #10, in order to validate the pathogenicity of their genotype findings, using anti-CD71 microbeads and positive selection through an AutoMACS separator (Miltenyi Biotec). A reticulocyte sample from patient 1 was prepared similarly, to be used as positive control. RNA was isolated using the QiaAmp RNA Blood Mini kit (QIAGEN) and reverse transcription was performed using the High-Capacity cDNA Reverse Transcription Kit (Applied Biosystems). *SPTA1* mRNA expression level was determined by qPCR using a StepOnePlus Real-Time PCR System (Applied Biosystems) and FAM-labeled Taqman probes (Thermo Fisher Scientific) for *SPTA1* spanning exons 48–49 (Assay ID Hs01005878_m1) and *ACTB* (Assay ID Hs01060665_g1) as a reference gene.

## Results and Discussion

We observed three different phenotypes in patients with recessive HS due to biallelic *SPTA1* mutations, verified to be *in trans* by parental targeted sequencing (Table 1). The heterozygous parents did not have any evidence of hemolytic anemia as expected, since even α^LELY^ *in trans* to a null *SPTA1* allele, producing a total of ∼25% of normal α-spectrin, causes no disease (Delaunay et al., 2004).

**FIGURE 1:**
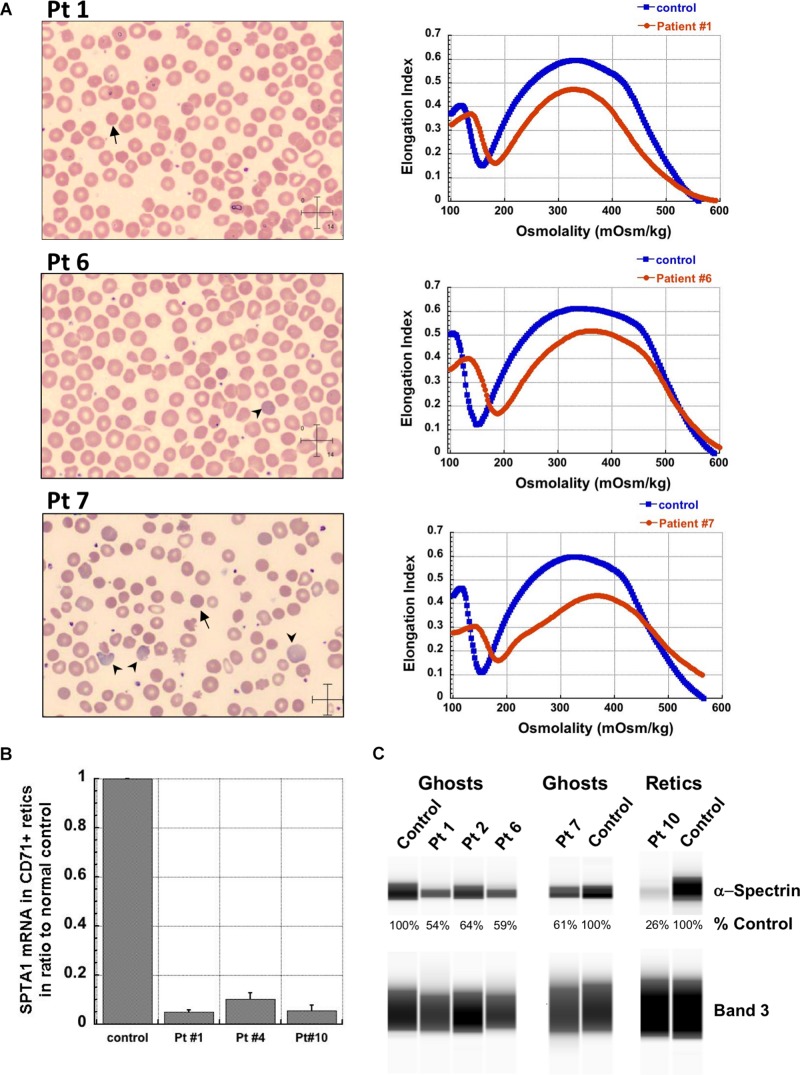
Studies in peripheral blood of patients with *SPTA1*-associated HS. **(A)** Peripheral blood smears on the left from patients 1, 6 and 7 showing multiple spherocytes (arrows) lacking central pallor due to decreased surface area to volume ratio and aniso-poikilocytosis. Patient 7 also has polychromasia with increased reticulocytes (arrowheads) indicating significant hemolysis prior to splenectomy. Ektacytometry, on the right, demonstrates the typical HS curve for the patients (red) vs. control (blue). HS is characterized by increased Omin indicating decreased RBC surface to volume ratio and decreased EImax (maximum Elongation Index) which depends mostly on the cytoskeleton mechanics. Frequently, the declining portion of the curve (represented by Ohyp, the osmolality value where the cells are at half of the maximum elongation) is also decreased (as in patient 1) indicating increased intracellular viscosity, however, it may also be normal as in patients 6 and 7 (Clark et al., 1983; Zaninoni et al., 2018). **(B)** qPCR in RNA isolated from patients’ reticulocytes demonstrated severely decreased α-spectrin expression. Patient 1 who has α^LEPRA^ *in trans* to a null *SPTA1* mutation was found to express α-spectrin at about 5% in comparison to normal control at levels similar to the original calculations for α^LEPRA^ (about 16% of full-length spectrin as compared to the normal *SPTA1* allele based on studies with metabolic labeling of erythroblasts *in vitro* (Wichterle et al., 1996), and therefore a total of 8% α-spectrin when α^LEPRA^ is *in trans* to a null allele). qPCR appears to be a useful diagnostic assay when an unknown low-expression allele is suspected *in trans* to a null *SPTA1* mutation or deletion in a disease suspected to be severe HS, such as the case of patient 4. **(C)** Quantitation of α-spectrin/band 3 ratio in RBC ghosts (Patients 1, 2, 6, and 7) and reticulocytes (Patient 10) expressed as percent of α-spectrin/band 3 ratio in corresponding normal control samples by immunodetection using size-based capillary electrophoresis. Agre et al. (1985) in one of the first descriptions of HS due to α-spectrin deficiency noted that the clinical severity of anemia was proportional to a degree of spectrin deficiency, ranging from 53% of normal spectrin content in severely anemic patients to 31% of normal in nearly lethal cases.

The first four patients listed in Table 1 (within Group I) had the well-known phenotype of *SPTA1*-associated HS, i.e., severe, transfusion-dependent HHA with brisk reticulocytosis (Agre et al., 1982; Wichterle et al., 1996). These patients presented with hyperbilirubinemia and non-immune hemolytic anemia soon after birth, requiring their first transfusion as neonates, before any meaningful RBC phenotypical testing could be obtained. They continued to require frequent transfusions, precluding any further erythrocyte phenotype work-up until splenectomy. Patients 1–3 had a *SPTA1* nonsense mutation (expected to lead to nonsense-mediated decay of the transcript) *in trans* to the low expression α^LEPRA^ mutation (c.4339-99C > T). Patient 1 required monthly transfusions for hemoglobin (Hgb) in the range of 6.4–7.5 g/dL with absolute reticulocyte count (ARC) of 130–360 × 10^9^/L for the first four years of life, with consequent iron overload, requiring chelation treatment with deferasirox. After molecular diagnosis of *SPTA1*-associated HS utilizing NGS of the HHA panel, he underwent partial splenectomy at 4 years of age. He was able to remain transfusion-free for 18 months with Hgb 8.7–11 g/dL, but then his anemia gradually worsened with increasing transfusion requirement. He had a follow-up total splenectomy at 7 years of age; at that time the remaining splenic tissue had been impressively regrown to 435 g, based on the pathology report. The patient has had no further transfusion requirement for the past 4 years, with Hgb now in 12.7–15.8 g/dL range. Blood smear from a non-transfused blood sample after splenectomy shows many spherocytes and moderate poikilocytosis, as expected with α-spectrin deficiency (Eber and Lux, 2004), and ektacytometry reveals the typical HS curve (Da Costa et al., 2016; Figure 1A). Patient 2 had a similar course for the first 2.5 years. After molecular diagnosis of *SPTA1*-associated HS, he underwent partial splenectomy and he remains transfusion-free since then with Hgb in the range of 9–10.8 g/dL and minimal regrowth of the remaining splenic tissue based on ultrasound (108–123 ml). Patient 3 has not yet had splenectomy due to parental preference and continues to require frequent transfusions.

Patient 4 had a similar clinical presentation but NGS for the HHA-panel was negative. Deletion/duplication assay by Comparative Genomic Hybridization was performed and identified a heterozygous deletion at 1q23.1 involving the *SPTA1* gene. No mutation was identified *in trans*. Since the patient remained transfusion dependent precluding phenotypical evaluation of his RBCs, α-spectrin mRNA expression in his reticulocytes was evaluated by qPCR and was found decreased at levels comparable to patient 1, confirming *SPTA1*-associated recessive HS (Figure 1B). Hgb normalized at ∼13 g/dL with no need for further transfusions post total splenectomy at 3 years of age.

Regrowth of the splenic remnant after partial splenectomy is a possibility, occasionally requiring a second surgery. Nevertheless, in both patients 1 and 2 who had splenectomy before 5 years of age in order to limit the transfusional iron overload, partial splenectomy was a reasonable choice with the goal to preserve splenic immune function (Englum et al., 2016). The different response of these two patients to partial splenectomy was most likely due to difference in splenic regrowth, rather than a difference in phenotypic severity. α-spectrin in the erythrocyte ghosts of patient 1 was determined by immunoelectrophoresis to be 54% vs. 64% for patient 2 in comparison to normal control (Figure 1C).

Surprisingly, we identified four patients (patients 5–8 in Table 1, comprising Group II) who, despite having equivalent genotype with the first four patients of a null *SPTA1* mutation *in trans* to α^LEPRA^, appeared to have a milder phenotype, ranging from improvement in transfusion requirement after frequent transfusions in the first 2–3 years of life (patients 5 and 6) to well compensated hemolysis (patient 8). The milder severity of the disease did not seem to correlate with RBC osmotic fragility or deformability based on ektacytometry (Figure 1A) or the α-spectrin quantitation in RBC ghosts which was only slightly lower compared to the α-spectrin level in patient 2 (Figure 1C). Additional gene polymorphisms and mutations found in the HHA panel (Supplementary Table S1) did not explain the milder phenotype noted in the patients of Group II versus Group I. Heterogeneity in the level of normal α-spectrin expression from the α-LEPRA allele (c.4339-99C > T), variability in HIF-pathway, and erythropoietin response, and sometimes a different tolerance to anemia by patients and/or parents may be contributing to differences in the phenotype between Group I and Group II patients.

The third phenotype (Group III in Table 1) associated with HS due to near-complete or complete α-spectrin deficiency is less well known since it has been typically embryonal lethal causing fatal *hydrops fetalis* in the third trimester of pregnancy or perinatally, such as the case of patient 9. His parents requested genetic counseling after having a fetus and a newborn child die with the clinical picture of *hydrops fetalis*. Both parents were found to carry a frameshift *SPTA1* mutation. DNA isolated from the patient’s liver tissue, preserved in paraffin after autopsy, was sequenced and revealed that the patient was compound heterozygous for these *SPTA1* mutations, predicting absence of normal α-spectrin production. With the progress of fetal medicine allowing prenatal diagnosis of severe anemia and *in utero* transfusions, more of these patients are now surviving to term and the disease is increasingly recognized.

Patient 10 was born prematurely at 33 weeks of gestation with severe non-immune hemolytic anemia requiring transfusion soon after birth. He remained transfusion dependent and total splenectomy did not significantly improve his hemolytic anemia. NGS for the HHA-panel revealed homozygosity for an intronic *SPTA1* variant, likely causing alternative splicing and severely decreased expression of α-spectrin, as verified by qPCR (Figure 1B) and immunoelectrophoresis performed in lysates prepared from isolated reticulocytes. The patient is now doing well after bone marrow transplant. Patient 11, an infant of Amish-Mennonite origin required five in-utero transfusions for severe anemia and had significant neonatal hyperbilirubinemia at birth requiring exchange transfusion. HHA gene panel revealed homozygosity for a *SPTA1* frameshift mutation, explaining her severe reticulocytopenia since her reticulocytes with complete α-spectrin deficiency are extremely fragile, failing to survive the shear stress in circulation.

The genetic and phenotypic information gathered from our patient cohort demonstrates that patients with recessive *SPTA1*-associated HS due to the low expression *SPTA1* variant α^LEPRA^ *in trans* to a null *SPTA*1 mutation respond to either partial or total splenectomy by significant improvement or resolution of anemia, respectively. In contrast, patients with near complete or complete α-spectrin deficiency because of biallelic null or rare intronic *SPTA*1 mutations that result in severely decreased expression of the protein, are unlikely to have a measurable response to splenectomy and require either lifetime transfusions and iron chelation or stem cell transplant. This group of patients have typically reticulocytopenia and history of having been salvaged from fatal *hydrops fetalis*, either because they were born prematurely and started transfusions early or because they had intrauterine transfusions.

Next generation sequencing panels are robust and rapid diagnostic tools for HHA, especially when frequent transfusions preclude phenotypic evaluation of the patients’ RBCs. Specialized assays such as qPCR of reticulocyte mRNA can be used for verification of the diagnosis. Comprehensive genetic and phenotypic evaluation is critical to provide insights into the variable phenotypes of patients with HHA and guide toward appropriate management.

## Ethics Statement

The manuscript is a retrospective case series, with no patient identifiable information, performed under two IRB-approved protocols in Cincinnati Children’s Hospital Medical Center (CCHMC) for diagnostic, non-interventional studies of Congenital Hemolytic and Dyserythropoietic Anemias: Study #2011-1510 and Study #2015-6150. Patients and families were consented either in person in CCHMC hematology clinic or over the phone in the presence of a witness.

## Author Contributions

SC, MR, KZ, WZ, and TK contributed to the conception and design of the study. SC, MR, HS, TM, ND, and KS performed the experiments. SC, ON, JR, LH, STC, JK, EK, MG, DW, and TK provided the clinical information. TK organized the database. SC and TK wrote the first draft of the manuscript. MR, HS, and KS wrote sections of the manuscript. All authors revised, read, and approved the submitted version of the manuscript.

## Conflict of Interest Statement

The authors declare that the research was conducted in the absence of any commercial or financial relationships that could be construed as a potential conflict of interest.
