# Corrigendum: The Spectrum of *SPTA1*-Associated Hereditary Spherocytosis

**DOI:** 10.3389/fphys.2019.01331

**Published:** 2019-10-18

**Authors:** Satheesh Chonat, Mary Risinger, Haripriya Sakthivel, Omar Niss, Jennifer A. Rothman, Loan Hsieh, Stella T. Chou, Janet L. Kwiatkowski, Eugene Khandros, Matthew F. Gorman, Donald T. Wells, Tamara Maghathe, Neha Dagaonkar, Katie G. Seu, Kejian Zhang, Wenying Zhang, Theodosia A. Kalfa

**Affiliations:** ^1^Department of Pediatrics, Emory University School of Medicine, Atlanta, GA, United States; ^2^Aflac Cancer and Blood Disorders Center, Children's Healthcare of Atlanta, Atlanta, GA, United States; ^3^College of Nursing, University of Cincinnati, Cincinnati, OH, United States; ^4^Cancer and Blood Diseases Institute, Cincinnati Children's Hospital Medical Center, Cincinnati, OH, United States; ^5^Department of Pediatrics, University of Cincinnati College of Medicine, Cincinnati, OH, United States; ^6^Duke University Medical Center, Durham, NC, United States; ^7^Division of Hematology, CHOC Children's Hospital and UC Irvine Medical Center, Orange, CA, United States; ^8^Division of Hematology, Children's Hospital of Philadelphia, Philadelphia, PA, United States; ^9^Department of Pediatrics, Perelman School of Medicine, University of Pennsylvania, Philadelphia, PA, United States; ^10^Kaiser Permanente Santa Clara Medical Center, Santa Clara, CA, United States; ^11^Dell Children's Medical Center, Austin, TX, United States; ^12^Genomics Analysis Facility, Institute for Genomic Medicine, Columbia University, New York, NY, United States; ^13^Coyote Bioscience Co., Ltd., San Jose, CA, United States; ^14^Laboratory of Genetics and Genomics, Division of Human Genetics, Cincinnati Children's Hospital Medical Center, Cincinnati, OH, United States

**Keywords:** *SPTA1*, α-spectrin, α^LEPRA^, hereditary spherocytosis, next generation sequencing, hemolytic anemia, *hydrops fetalis*

In the original article, there was a mistake in Table 1 as published. The *SPTA1* mutation of Allele 2 in Patient 1, is stated as “c.4294T>A (p.L1432^*^).” The correct mutation should read “c.4295del (p.L1432^*^).” The corrected Table 1 appears below.

**Table 1:** Genetic mutations and associated phenotype in HS due to SPTA1 mutations.

**Phenotype**	**Patient**	**Allele 1**	**Allele 2**	**Age at time of report and comments**	**Ektacytometry**	**α-spectrin in RBC ghosts (% of control)**
GROUP I (patients 1–4) Severe, recessive HS (transfusion-dependent, responding to splenectomy)	1	c.4339-99C > T	c.4295del (p.L1432^*^)	11 year-old, chronic transfusion requirement with partial response to partial splenectomy, resolved after total splenectomy	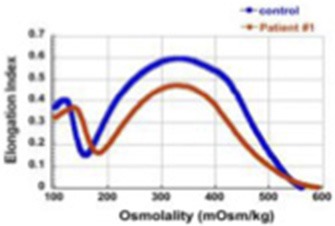	54%
	2	c.4339-99C > T	c.5102A > T (p.L1701^*^)	7 year-old, chronic transfusion requirement, improved with partial splenectomy	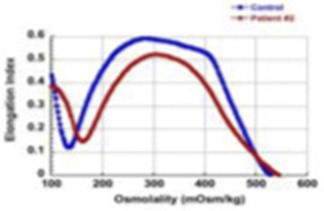	64%
	3	c.4339-99C > T	c.3267A > T (p.Y1089^*^)	11 year-old, not splenectomized due to family preference, continues to require frequent transfusions	Not evaluable in a transfused sample	
	4	Mutation not identified	Gross deletion of *SPTA1*	3.5 year-old, RT-PCR demonstrated significantly decreased α-spectrin expression; hemoglobin has normalized after recent splenectomy	Not evaluable in a transfused sample	
GROUP II (patients 5–8) Severe to moderately severe, recessive HS	5	c.4339-99C > T	c.1120C > T (p.R374^*^)	4 year-old, chronic transfusion requirement for first three years with improved pattern since.	Sample not provided after age 3, when transfusion-independent	
	6	c.4339-99C > T	c.1351-1G > T	7 year-old, occasional transfusion requirement, resolved after splenectomy at 5 years of age	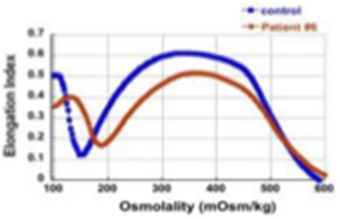	59%
	7	c.4339-99C>T	c.2671C > T (p.R891^*^)	4 year-old, has not been transfused so far, Hgb 7.1-8.9 g/dL, ARC 420-572 x 10^3^/μl.	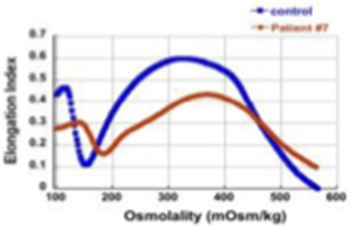	61%
	8	c.4339-99C > T	c.3257delT	8 year-old, transfused once as neonate, Hgb 10.6–11.8 g/dL, ARC 354–535 x 10^3^/μl; now Hgb 15–16 g/dL with normal ARC after splenectomy at 6 years of age (splenectomy performed because of chronic abdominal pain due to co-morbidities)	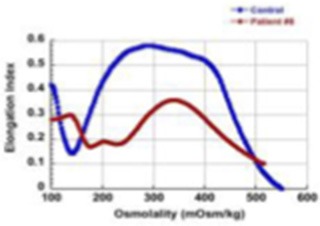	Not performed.
GROUP III (patients 9-11) Life-threatening anemia in utero leading to fatal *hydrops fetalis* if untreated (transfusion-dependent, not responding to splenectomy)	9	c.4206delG (fs)	c.4180delT (fs) in haplotype with c.6631C > T (p.R2211C)	Died at birth. Post-mortem diagnosis from parental studies and DNA extracted from liver tissue saved in paraffin block	N/A	
	10	c.6788+11C > T	c.6788+11C > T	11 year-old, born prematurely at EGA of 33 weeks with *hydrops fetalis*, remained transfusion-dependent even after splenectomy; now doing well after matched sibling transplant	Not evaluable in a transfused sample (required chronic transfusions up until bone marrow transplant)	26% (performed in CD71+ cells)
	11	c.6154del (p.Ala2052fs)	c.6154del (p.Ala2052fs)	2 year-old, severe in-utero anemia requiring five *in-utero* transfusions. Born with severe neonatal hyperbilirubinemia requiring exchange transfusion. Remains transfusion-dependent	Not evaluable in a transfused sample	

Of note, all the SPTA1 variants reported here except c.4339-99C > T (α^LEPRA^) and c.2671C > T; p.R891

* *(Bogardus et al., 2014) have not been previously described*.

The authors apologize for this error and state that this does not change the scientific conclusions of the article in any way. The original article has been updated.
