# Supplementary material for: The Spectrum of SPTA1-Associated Hereditary Spherocytosis
Source: Front Physiol. 2019 Jul 3;10:815. doi: 10.3389/fphys.2019.00815 (PMC6617536; doi:10.3389/fphys.2019.00815)
Supplement: Supplementary file 1 [file Data_Sheet_1.PDF]

Supplemental Table: Additional pathogenic variants and variant of unknown clinical significance identified with the HHA panel

Patient	Gene	Refseq and variant	Amino acid change	Zygosity	Interpretation	HGMD	CLinVar	gnomAD-All	in sillico prediction
2	*KLF1*	NM_006563.3:c.604G>A	p.Gly202Arg	het	VUCS	not reported	not reported	0.0064%	pathogenic
	PKLR	NM_000298.5:c.1456C>T	p.Arg486Trp	het	likely pathogenic	DM	path/likely path	0.2900%	pathogenic
	SPTA1	NM_003126.3:c.803G>A	p.Arg268Gln	het	VUCS	not reported	not reported	0.0007%	conflicted
8	*G6PD*	NM_001042351.2:c.844G>C	p.Asp282His	hemi	Pathogenic (class III G6PD variant)		pathogenic		
10	*PIEZO1*	NM_001142864.2:c.4674C>T	p.Gly1558Gly	HET		not reported	not reported	0.0330%	not predicted to create or disrupt a splice site
11	*ABCG5*	NM_022436.2:c.1535T>C	p.Ile512Thr	HET	VUCS	not reported	not reported	0.0028%	conflicted
	ABCG5	NM_022436.2:c.969G>T	p.Gln323His	HET	VUCS	not reported	not reported	0.0004%	benign
	EPB42	NM_000119.2:c.520+12C>T		HET	VUCS-LB	not reported	VUS	0.1400%	not predicted to create or disrupt a splice site
	PIEZO1	NM_001142864.2:c.1356C>G	p.Leu452Leu	HET	VUCS	not reported	not reported	NA	not predicted to create or disrupt a splice site
	SEC23B	NM_006363.4:c.2202T>C	p.Tyr734Tyr	HET	VUCS	not reported	not reported	NA	may disrupt a splice donor
